# CD20^+^ T cells: an emerging T cell subset in human pathology

**DOI:** 10.1007/s00011-022-01622-x

**Published:** 2022-08-11

**Authors:** Adrian Y. S. Lee

**Affiliations:** 1 Department of Clinical Immunology, Westmead Hospital, Hawkesbury Road, Westmead, NSW 2145 Australia; 2 Department of Immunopathology, ICPMR and NSW Health Pathology, Westmead Hospital, Westmead, NSW Australia; 3 Department of Medicine, Westmead Clinical School, The University of Sydney, Westmead, NSW Australia

**Keywords:** CD20, Cellular immunology, Flow cytometry, *T* cells

## Abstract

**Introduction:**

Although CD20 is classically a *B* cell marker, in the last three decades, dim expression has been noted on a subset of *T* cells as well that has been independently verified by a number of groups. Our understanding of these cells and their function is not well established.

**Methods:**

A thorough review of original articles on CD20^+^ *T* cells was undertaken of Pubmed by using combination of phrases including “CD20^+^”, “CD20-positive” and “T cells”. Articles in English were considered, and there was no time restriction.

**Results:**

CD20^+^ *T* cells express the standard T cell markers and, in comparison to CD20¯ T cells, appear to express greater inflammatory cytokines and markers of effector function. Although the ontogeny of these cells is still being established, the current theory is that CD20 may be acquired by trogocytosis from *B* cells. CD20^+^ *T* cells may be found in healthy controls and in a wide range of pathologies including autoimmune diseases, haematological and non-haematological malignancies and human immunodeficiency virus (HIV) infections. One of the best studied diseases where these cells are found is multiple sclerosis (MS) where a number of therapeutic interventions, including anti-CD20 depletion, have been shown to effectively deplete these cells.

**Conclusion:**

This review summarises the latest understanding of CD20^+^ *T* cells, their presence in various diseases, their putative function and how they may be an ongoing target of CD20-depleting agents. Unfortunately, our understanding of these cells is still at its infancy and ongoing study in a wider range of pathologies is required.

## Immunobiology of CD20^+^*T* cells

### Introduction

Cluster of differentiation 20 (CD20) is a lymphocyte surface molecule that has become a ubiquitous marker for *B* cells in flow cytometry, being expressed on pre-*B* cells to mature *B* cells, and lost in plasmablasts and plasma cells [1]. It is a molecule that ranges from 33 to 37 kDa and is encoded by the *MS4A1* gene located on chromosome 11 [1]. Functionally, it is thought to serve as a calcium channel [2] and is important in regulating *B* cell function by associating with the *B* cell receptor [3].

Since the 1990 s, observations were made, by flow cytometry, that a small percentage of human CD3^+^ *T* cells in peripheral blood dimly express CD20 on their surface [4]. This is in contrast to the traditional bright expression of CD20 found on *B* cells which are approximately 15-fold brighter by flow cytometry [5]. CD20^+^ *T* cells may range from 1 to 6% of *T* cells in peripheral blood (PB) of healthy controls (HCs), and do not express any other markers of *B* cells such as CD19 [6, 7]. The frequency of these cells bear no relationship to age of patient, sex or CD4:8 ratio [8, 9]. They may also be found in other human tissues such as the thymus, tonsils, bone marrow and cerebral spinal fluid (CSF) [6, 10].

However, the appearance of CD20 on *T* cells has generated some controversy. Some maintain that part of the CD20 expression on *T* cells may be a product of *T*-*B* cell doublets. As evidence for this, ethylenediaminetetraacetic acid treatment of PB reduces the frequency of CD20^+^ *T* cells, as does the exclusion of doublets via forward and side scatter gating [9]. However, dim expression of CD20 create a distinct subset from the bright CD20 found on B cells and furthermore, these cells lack CD19, effectively ruling them out as *T*-*B* cell doublets [7, 11]. Multiple anti-CD20 clones have reproduced similar results and blocking studies have revealed that CD20^+^ *T* cells are not a product of non-specific binding of these anti-CD20 clones [4]. Nevertheless, they are, to this day, recognised as a bona fide subset that has been independently verified by a large number of groups and using other techniques such as single-cell imaging flow cytometry and immunohistochemistry [12, 13].

### Origins

The origin of CD20^+^ *T* cells is not entirely clear. Certainly, these cells express *MS4A1* mRNA suggesting an intrinsic ability to generate CD20 protein [10]. In monkey experiments, isolated CD20^+^ *T* cells up-regulate *MS4A1* mRNA and surface CD20 expression after concanavalin A and interleukin (IL)-2 stimulation in vitro indicating that expression may also be regulated by the cells’ microenvironment [14].

CD20 protein expression is absent on *T* cells of human cord blood, suggesting acquisition of this molecule later in development [6]. In mice, CD20^+^ *T* cells only appear in the spleen after week 2 of age supporting this notion [15]. By co-culturing *T* and *B* cell lines, de Bruyn et al. [6] were able to show an increase in CD20 expression on *T* cells after just 1 h. They speculated that the mechanism of this was via trogocytosis when *T* cells make contact with their cognate *B* cells [6]. This theory was proven more recently when co-culturing experiments of mice lymphocytes revealed that direct contact with *B* cells was necessary for the transference of CD20 to *T* cells [15]. However, why they are only selective for CD20 and not any other *B* cell surface markers is not clear. A later study performed transcriptomic analysis via RNA sequencing revealing activated CD20^+^ *T* cells derived from HCs do not upregulate *MS4A1* supporting de Bruyn’s theory of trogocytosis [5].

### Phenotype

After the exclusion of doublets, CD20^+^ *T* cells are commonly identified via flow cytometry as CD3^+^CD19¯ lymphocytes with dim expression of CD20. They may express CD4, CD8 or neither; but are particularly enriched with CD8 compared to CD20¯ *T* cells in HCs [5]. HCs may have around three-quarters of their PB CD3^+^CD20^+^ compartment as CD8^+^ *T* cells [5, 16], In multiple sclerosis (MS) patients, similar percentages are also seen [17].

Human CD20^+^ *T* cells may be either HLA-DR positive or negative, and may express either CD45RA or CD45RO [4, 6, 10], suggesting a differential role for these cells. In MS patients, CD4^+^ and CD8^+^CD20^+^ *T* cells contain higher central and effector memory *T* cells over CD20¯ *T* cells [15]. Whilst CD69 is generally absent on CD20^+^ *T* cells in HCs, in a disease model (psoriasis), there is marked up-regulation of surface CD69 indicating activation of these cells [18].

They are more likely to express γδ *T* cell receptors over their CD20¯ *T* cell counterparts, as assessed in HCs [4]. Assessment of chemokine receptor profiles in the PB of MS patients reveal that CD20^+^ *T* cells bear CC chemokine receptor (CCR)2, CCR5, CCR6, CXC chemokine receptor 3 (CXCR3) and CXCR6, and more so than CD20¯ *T* cells [19, 20]. In HCs and psoriasis, around half of the CD20^+^ and CD20¯ *T* cells are CCR7^+^.[18] Other phenotype markers include CD49d^+^ [20], CD27^+^, CD28^+^ and a smaller fraction express CD38 [18]. They are negative for CD40 [18]. These data indicate that in human disease, CD20^+^ *T* cells are likely a more active subset of cells and display a rich diversity of chemokine receptors to assist with CD20^+^ *T* cell homing to target organs.

### Function

Although the function of these cells, and their roles in the immunopathogenesis of various diseases, are still being unearthed, it is clear that CD20^+^ *T* cells play an important effector role. Compared to CD20¯ *T* cells, the CD20^+^ *T* cells express significantly greater IL-2, IL-4, IL-10 IL-17, tumour necrosis factor (TNF), interferon (IFN)-γ and granulocyte macrophage colony-stimulating factor (GM-CSF) as assessed flow cytometrically [7, 10, 17, 20]. These were seen in both HCs and diseased patients (e.g., MS) indicating that these cells express inflammatory cytokines constitutively. Interestingly, sorted and stimulated CD20¯ from RA patients secreted more IL-2 and IL-10 (by bead immunoassays), however, [7]. The variety of cytokines hints at a large variety of inflammatory and immunomodulatory functions of these cells. Furthermore, and in line with their CD8^+^ expression, granzyme and perforin expression has been noted in patients with MS, indicating a cytotoxic potential for these cells [21].

## Role in autoimmunity

### Multiple sclerosis (MS)

By far the most studied disease with these cells is MS, a devastating demyelinating disorder of the nervous system. Observation studies identify the presence of these cells in the PB, CSF [20] and brain white matter of patients with MS [19]. Increased percentage of *T* cells of both CD4^+^ and CD8^+^ are noted in the PB of MS patients (approximately 5–15%) compared to HCs [20]. However, other studies found no differences in this percentage even after stratifying the cells for CD4 and CD8 expression [10, 22]. It is possible that heterogeneity of patients and different anti-CD20 clones have contributed to these apparent contradictory findings.

In brain biopsies of MS patients, the *T* cells are predominantly CD8^+^ tissue resident memory (*T*_RM_) cells that constitutively express dim CD20 and *MS4A1* mRNA [19]. Increased density of these CD20^+^ *T* cells were found in MS white matter active lesions compared to HC patients. These cells had a higher expression of CXCR6, Ki-67 and granzyme B than CD20¯CD8^+^ *T*_RM_ cells [19]. Both CD4^+^ and CD8^+^ CD20^+^ *T* cells co-localised with IL-17 on immunohistochemistry and secreted IFN-γ on flow cytometry [22], confirming their pro-inflammatory function. These circumstantial data hint that in MS, CD20^+^ *T* cells are pro-inflammatory and possibly contribute to the neuropathology.

More elaborate translational research has found myelin-specific CD8^+^ *T* cells in the PB of untreated MS patients, using tetramer constructs [23]. Around 10% of these cells expressed CD20 and in line with Hsiao et al.’s study [19], were predominantly a memory phenotype (CD45RA¯) [23]. As a proof of principle of their pathogenicity, other researchers have utilised experimental autoimmune encephalomyelitis (EAE) mice (murine model for MS) and adoptively transferred murine CD20^+^ *T* cells, using CD20¯ *T* cells as the comparator. Mice who had the former developed worse clinical and histological disease, supporting the above circumstantial evidence for their pathogenicity [15].

The role that therapeutic anti-CD20 agents have in MS is well recognised [24], hinting at the role that *B* cells play in the pathogenesis of this disorder. It is reasonable to assume that given the putative role that CD20^+^ *T* cells have in MS pathogenesis, that CD20 depletion functions in part by targeting such cells. Indeed, it has been shown that anti-CD20 therapy (such as rituximab and ocrelizumab) effectively depletes CD20^+^ *T* cells in both the PB and CSF of patients with MS [10, 25]. Onset of depletion can be as quick as 2 weeks and has been seen up to 6 months post administration [26, 27], and CD8^+^CD20^+^ *T* cells appear to somewhat more susceptible to CD20 depletion over CD4^+^CD20^+^ *T* cells [12]. However, it is important to realise that a depletion in CD20^+^ *T* cells or B cells are not the only mechanisms for the efficacy of CD20-depleting agents, as an increase in PB regulatory *T* (T_Reg_) cells after ocrelizumab administration was seen, which may assist with the dampening down of neuroinflammation [28].

Other therapeutic options have been shown to modulate CD20^+^ *T* cell numbers as well. Compared to untreated MS patients, alemtuzumab, dimethyl fumarate and fingolimod therapy decreased the absolute numbers of CD20^+^ *T* cells whilst natalizumab increased these, suggesting that CD20^+^ *T* cells enter the CNS via an α4-integrin-dependent manner [10].

### Rheumatoid arthritis (RA)

The role that *T* cells play in the pathogenesis of RA remains well recognised and includes “classic” *T* cells such as *T* helper 17 (T_H_17) cells [29]. Interest in CD20^+^ *T* cells and RA, therefore, has emerged in the last decade. When looking at the proportions of circulating CD20^+^ *T* cells in PB, these number approximately 1–2% of *T* cells in RA patients and are comparable to HCs [7, 16]. Similarly to CD20^+^ *T* cells in MS, these are effectively depleted by rituximab, in part, related to their susceptibility to apoptosis [7].

However, when CD20^+^ *T* cells are stratified on their ability to secrete IL-17 (approximately 24% of IL-17-secreting *T* cells), there was a marked increase in the percentage of these cells over HCs—some 240-fold increase in median percentage [16]. This implies that some *T*_H_17 cells, which have often defined as a *T* cell that secretes IL-17, are in fact, CD20^+^.

Furthermore, whilst rituximab does little to affect the overall *T* cell numbers in the PB pre- and post-therapy [30], one study found that rituximab selectively depleted *T*_H_17 cells in the synovium of RA patients [31], which similar to the MS studies, may be the result of their CD20 positivity. However, this could also be a result of *B* cell depletion rather than a direct effect on *T*_H_17 cells, particularly because only a limited number of these cells express CD20. A further mechanism may be the alterations of cytokine environment and consequential skewing towards *T*_reg_ cells following rituximab therapy [32].

### Psoriasis

The role of *T* cells and their inflammatory cytokine milieu in the pathogenesis of psoriasis is also well recognised [33]. In the PB, 1–2% of lymphocytes are CD20^+^ *T* cells which were comparable in proportion to HCs [18]. However, when these cells were further stratified to the expression of CD4 or CD8, only CD20^+^ *T* cells that expressed CD4 and/or CD8 were elevated; only double negative CD20^+^ *T* cells were comparable to HCs [34]. This interesting observation is analogous to some of the MS studies where differences in CD20^+^ T cell proportions were only seen upon CD4/CD8 stratification [20].

Like the aforementioned diseases, CD20^+^ *T* cells are thought to be inflammatory and contribute to the disease pathogenesis. In contrast to HCs and CD20¯ *T* cells, psoriasis patients’ CD20^+^ *T* cells were enriched with CD4^+^ *T* cells, displayed an activated phenotype (CD69) and secreted greater IFN-γ, TNF and IL-21 as assessed flow cytometrically [18]. Double negative CD20^+^ *T* cells were also found to be inversely related to disease duration and disease severity, giving some clinical utility to measuring these in the diagnostic laboratory [34]. Indeed, circulating double negative *T* cells have received special attention for their purported role in the pathogenesis of psoriasis [35]; however, this study did not look at the CD20 expression on these cells.

### Sjögren’s syndrome

Only one investigation regarding CD20^+^ *T* cells could be found in the systemic autoimmune diseases. Like the autoimmune disorders above treated with rituximab, double negative IL-17^+^ *T* cells, as opposed to other *T* cell subsets, were suppressed by approximately 50% 3 months following rituximab therapy in patients with Sjögren’s syndrome [36]. Examination of these cells revealed approximately 29% of these expressing CD20. Hence, it is likely that other mechanisms apart from CD20 depletion is at play in the targeted reduction of these IL-17^+^ cells. The role for these CD20^+^IL-17^+^ *T* cells in Sjögren’s syndrome was not explored but it is probable that given their IL-17 secretion, they likely remain a pathogenic subset of cells. In in vitro models of Sjögren’s syndrome, human salivary glands treated with IL-17 increased cell death suggesting a direct pathogenesis for these IL-17^+^ *T* cells in contributing to salivary gland dysfunction [37].

## Role in malignancies

### Lymphomas

A large range of CD20^+^ *T* cell lymphomas has been described in the literature. The exact frequency of such is unclear as a detailed epidemiological study has not been performed; however, it is thought to be quite rare. They tend to occur in elderly and male patients [38]. These lymphomas may be CD4^+^, CD8^+^ or double positive [39], and it is not clear if the lymphoma arises from normal CD20^+^ *T* cell precursors, or develop CD20 expression aberrantly. Although the literature is focused on primary CD20^+^ *T* cell lymphomas, these cells have occasionally been described in mediastinal [39] and pleural [40] *B* cell lymphomas. This gives rise to the possibility that these cells may be reactive to the tumour and may exert anti-tumour immunity; although no evidence for the latter currently exists. One interesting case on a CD20^+^ *T* cell lymphoma documented CD20 expression on malignant *T* cells nodally whilst the patient’s skin lesions lacked this marker [41]. This probably reflects clonal evolution of the tumour and indicates that CD20 itself is a dynamic marker that may either be gained or lost. It may also relate to the cytokine microenvironment since cytokines may modulate CD20 expression on CD20^+^ *T* cells in in vitro monkey experiments [14].

Primary CD20^+^ *T* cell lymphomas have been described nodally or extra-nodally. Described extra-nodal sites have included the nose [38], neck [42], skin [43], and gastrointestinal tract [44]. Generally, these CD20^+^ *T* cell lymphomas express pan-*T* cell markers, confirming their identity as *T* cells, although cases do exist with the aberrant loss of normal *T* cell markers [45]. The usual method of identification is via immunohistochemistry and flow cytometry. However, given the rarity of CD20^+^ *T* cell lymphomas, and aberrant expression of lymphocyte markers, some caution is needed to avoid a misdiagnosis of a *B* cell lymphoma [41]. B cell expansion in response to a primary *T* cell leukaemia/lymphoma may also complicate the diagnosis [46].

Given their CD20 expression, the use of rituximab and other CD20-depleting agents for therapy remains a possibility. A review by Kakinoki et al. qualitatively found that CD20 staining intensity roughly correlated with the cancer’s response to therapy, with *T* cell lymphomas that had stronger CD20 expression appearing to enter remission more readily compared to others that displayed weak or variable CD20 expression [47]. This gives some justification to reporting CD20 staining intensities semi-quantitatively in these group of patients.

### Leukaemias

Several primary CD20^+^ mature and immature *T* cell leukaemias have been described in the literature, which may be part of the same disease spectrum as *T* cell lymphomas. Similar to lymphomas, they may be CD4^+^, CD8^+^ or double positive [48, 49]. Case series on CD20^+^ *T* cell lymphoma/leukaemias (ATLL) show that they may occur in children or adults, tend to be associated with an aggressive course and can be associated with human T-lymphotropic virus (HTLV) [49, 50]. In contrast, a case of a CD20^+^ *T* cell large granular lymphocytic leukaemia associated with RA had a good prognosis with no specific therapy [48]. Patients have received conventional chemotherapy but no case of the use of anti-CD20 therapy could be found. Being a unique marker on *T* cells, CD20 may be a useful marker for performing minimal residual disease analysis by flow cytometry.

### Non-haematological malignancies

There are only limited studies looking at CD20^+^ *T* cells in non-haematological malignancies. In tissue studies, tumour-infiltrating lymphocytes (TILs) are well studied in the literature and are stratified using immunohistochemical methods into *T* and *B* cells [51]. For the latter, CD20 is oftentimes used as a sole *B* cell marker which unfortunately, may lead to incorrect attribution of all CD20^+^ lymphocytes as being *B* cells. Future studies would need to delineate the nature of these cells in various malignancies.

Circulating CD20^+^ *T* cells, on the other hand, have been explored in a few studies. In a group of patients with ovarian cancer, these cells were detectable in the PB of patients, at approximately 6% and not differing significantly from HCs [6]. They were also found in the ascitic fluid of these patients, were found to be predominantly CD8^+^ effector memory *T* cells, and secreted IFN-γ [6]. Compared to the PB of ovarian cancer patients, the CD20^+^ *T* cells were enriched in the ascitic fluid (23% vs. approximately 6% in the PB) suggesting a tumour-specific function of these cells [6]. *T* cell expansion in response to tumours are well recognised and may be a bystander phenomenon or exert anti-tumour responses [52]; it is probable that CD20^+^ *T* cells may also behave similarly.

In a study of colorectal cancer, CD20 (*MS4A1*) expression was noted on PB and tumour *T* cells, respectively [53]. CD8^+^ *T* cells in the tumour, compared to corresponding cells in non-neoplastic colonic tissue, downregulated CD20 expression. Nivolumab, a programmed cell death-1 (PD-1) blocker, was then used to explore a possible mechanism for *MS4A1* modulation. Interestingly, nivolumab-bound *T* cells had higher expression of *MS4A1* than unbound *T* cells suggesting a mechanism for downregulation of CD20 in tumour-associated *T* cells is via the PD ligand 1-PD1 pathway [53].

## Role in infections

### Human immunodeficiency virus (HIV)

CD20^+^ *T* cells have been found in the bone marrow and PB of patients with HIV [8, 54]. In the PB of untreated HIV patients, there is a reduction in the proportion of circulating CD20^+^ *T* cells (percent of lymphocytes) compared to HCs, which is somewhat restored following treatment [54]. However, stratifying these cells by CD4 yields conflicting results in the literature depending on how their frequency is defined. Förster et al. [54] found no difference in the CD4^+^CD20^+^ *T* cells expressed as a percentage of lymphocytes across treated and untreated patients, and HCs. They did, however, find a relative increase in CD8^+^CD20^+^ *T* cells in untreated patients compared to treated patients and HCs [54]. In contrast, when CD4^+^CD20^+^ *T* cells were expressed as a percentage of CD4^+^ *T* cells, Serra-Peinado et al. [11] found untreated HIV patients had a higher proportion of CD4^+^CD20^+^ *T* cells over treated HIV and HCs (at approximately 1% of CD4^+^ *T* cells). Phenotypically, these CD20^+^ *T* cells had a more activated phenotype than CD20¯CD4^+^ *T* cells with greater expression of CD69 and HLA-DR, and were predominantly memory cells (CD45RO^+^) [11], in line with activated CD20^+^ *T* cells in MS (see above). However, the literature on these cells in HIV is still preliminary and it is unclear what role they play in the pathogenesis or immunity against HIV.

The question remains if these CD4^+^CD20^+^ *T* cells may act as viral reservoirs. In HIV patients, regardless of treatment status, higher HIV RNA and DNA was noted in CD4^+^CD20^+^ over CD4^+^CD20¯ *T* cells [11, 55]. It is possible that HIV may, hence, cause activation of these cells and CD20 up-regulation. Similarly, in the animal models for HIV (SIV and African green monkeys and macaques), CD20 (along with CD32) identified CD4^+^ *T* cells that contained higher actively transcribed SIV RNA over CD32¯CD4^+^ *T* cells [56]. Serra-Peinado et al. examined the effects of the addition of rituximab to co-cultures of these CD4^+^CD20^+^ cells and found that they were efficiently targeted by this drug and completely abrogated HIV replication [11]. These data indicate that CD20 is a reliable marker of HIV-activated CD4^+^ *T* cells and CD20-depleting agents to target these virus-infected cells may be a therapeutic option in the future.

### Other infections

Apart from HIV, there has been very little in the literature examining the role and presence of CD20^+^ *T* cells in infections. In a clinical, observational study of patients with adenovirus pneumonia, higher absolute numbers of CD4^+^, CD8^+^ and CD20^+^ *T* cells were noted in the PB of patients without pleural effusion, compared to those patients with [57]. Whilst the mechanism is unclear, the authors opined that viral suppression of T cells in a more extensive disease (with effusion) may be responsible for the finding [57].

## Discussion and conclusion

**Fig. 1:**
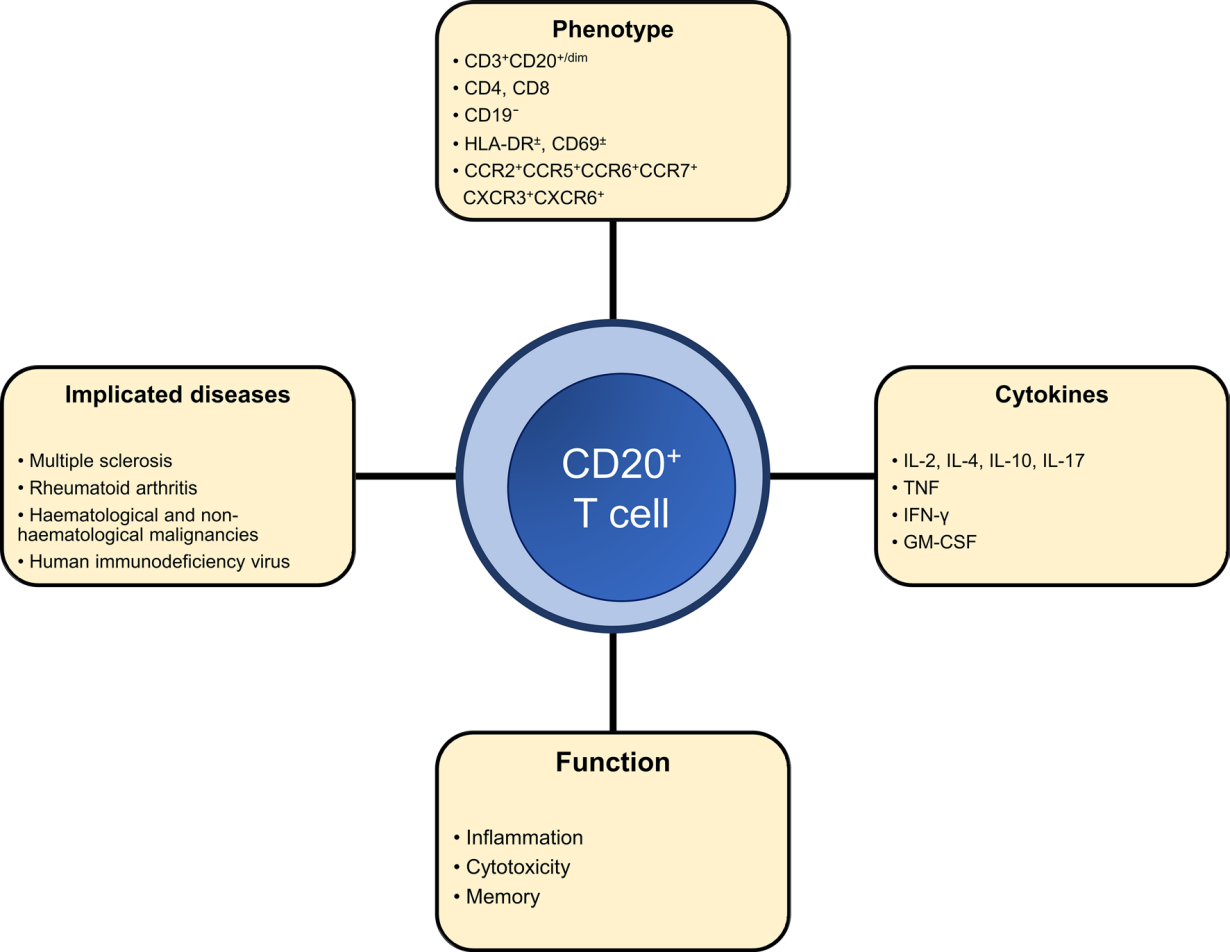
Key features of CD20^+^ *T* cells. *IL* interleukin, *TNF* tumour necrosis factor, *IFN* interferon, *GM-CSF* granulocyte–macrophage colony stimulating factor

Although still in the infancy of our understanding of the role and function of CD20^+^ *T* cells, it is clear that they are emerging as a *T* cell subset of interest that is involved in a variety of pathologies. Figure 1 summarises some of the important features of CD20^+^ *T* cells presented in this article. With the recognition of CD20 on *T* cells, older studies that use this as a sole marker of *B* cells in conditions such as lymphomas and cancers (TILs) should prompt some care in the interpretation of results. At this point in time, it is unclear whether these cells are reactive, are directly pathogenic or both. Only the role they play in MS has been studied in some detail where recent studies are suggesting that CD20^+^ *T* cells are pathogenic. Unfortunately, there is a paucity of detailed studies examining their role in the pathogenesis of other diseases and most studies are currently restricted to being just observational studies.

The most basic observations of the circulating frequency of CD20^+^ *T* cells is fraught with uncertainty and heterogeneity as studies use various definitions of defining the parent populations. However, the mere quantitation of cells reveals little about their function. Nevertheless, the presence of CD20^+^ *T* cells in HCs and evidence for their function through cytokine secretions suggests that they play a role in providing “background” immunity or homeostasis.

In addition, the relationship and function between their CD20¯ *T* cell counterparts need exploring. Given that CD20^+^ *T* cells comprise only a fraction of *T* cells, and represent a more “activated” and inflammatory phenotype over CD20¯ *T* cells, it is possible that CD20¯ *T* cells represent cells that are resting or providing homeostatic functions. The strong proliferative capabilities in CD20¯ *T* cells over CD20^+^ [7] indicates that these cells are likely poised to become effector cells. However, these notions are not solidly proven in the available literature and further work is required to elucidate the role and association of this subset.

Immune cells often work in concert with each other to drive pathology; however, the interactions and relationships of these cells with other immune cells has been poorly studied. From the limited literature so far, it is probable that CD20^+^ *T* cells interact with their cognate *B* cells which facilitates CD20 trogocytosis to the receiving *T* cell. CD20^+^ *T*-*B* cell conjugates have been identified in situ in tonsillar tissue confirming the interaction beyond in vitro experiments [6]. This likely leads to antigen presentation, subsequent *T* cell activation and the differentiation into memory and effector subsets.

A further avenue for exploration is the IL-17-secreting properties of CD20^+^ *T* cells and the notion that these represent a subset of *T*_H_17 cells. Overall, less than 5% of CD20^+^ *T* cells secrete IL-17 [10]. *T*_H_17 cells have overwhelmingly been implicated in the pathogenesis of several autoimmune disorders including MS and RA [58]. *T*_H_17 cells exert their pathogenicity through IL-17 and GM-CSF cytokines [58], which are also some the key cytokines secreted by the CD20^+^ *T* cell subset. Only around a quarter of IL-17-secreting *T* cells express CD20, [16, 36] making them a subset that likely overlaps with classic *T*_H_17 cells. How these CD20^+^ cells are different phenotypically and functionally from the CD20¯ IL-17-secreting *T* cells is not clear and no published research is yet available to answer this question. IL-17 production in CD4^+^ *T* cells, nevertheless, appears to be predominantly restricted to the CD20^+^ subset [17]. Additionally, it is not clearly if the IL-17-secreting CD20^+^ *T* cells is also programmed by the classical retinoid orphan receptor gamma t (RORgt) master transcription factor seen in *T*_H_17 cells.

Hence, there is certainly an urgent clinical need to study these cells, particularly with the availability of anti-CD20 therapeutic agents which could deplete these cells. Future studies should explore the role and function of these cells in other diseases such as other non-haematological malignancies and infections. Some important areas to explore would include whether other *T* cell subsets may express CD20 and if so, do they signify a more “immunologically active” subset with greater effector functions? Furthermore, it would be interesting to see of the enumeration of CD20^+^ *T* cells in various diseases offer any useful diagnostic, prognostic or predictive potential in selected diseases. Indeed, we have only scratched the surface in our understanding of this enigmatic subset and modern research techniques such as single-cell RNA sequencing should surely shed light on these relatively unexplored cells.
